# The role of the baroreflex and parasympathetic nervous system in fructose-induced cardiac and metabolic alterations

**DOI:** 10.1038/s41598-018-29336-3

**Published:** 2018-07-20

**Authors:** Fernando dos Santos, Ivana C. Moraes-Silva, Edson D. Moreira, Maria-Claudia Irigoyen

**Affiliations:** Heart Institute (InCor), School of Medicine, University of Sao Paulo (FMUSP), Sao Paulo, Brazil

## Abstract

It is well-established that baroreflex sensitivity is essential for blood pressure control, and also plays a key role in the modulation of disease-induced metabolic alterations. In order to investigate the role of the baroreflex in the cardiometabolic and inflammatory derangements promoted by fructose overload, Wistar rats underwent sinoaortic denervation (SAD) or sham surgery and were studied 90 days after receiving tap water (Den and Ctrl) or a 10% fructose solution (Fruc and Den-Fruc). All experimental groups showed marked and similar degree of baroreflex impairment compared to Ctrl. As expected, fructose overload effectively induced metabolic syndrome; however, when it was associated with SAD, several alterations were attenuated. While Fruc rats displayed increased sympathetic modulation and tone and reduced vagal modulation compared to Ctrl animals, Den-Fruc rats showed greater vagal tone and modulation when compared to the Fruc group. Moreover, the Den-Fruc group showed augmented expression of β1 adrenergic receptors and TNF/IL-10 ratio and reduction of β2 in the left ventricle. The increase in vagal function was correlated with improved insulin sensitivity (r^2^ = 0.76), and decreased abdominal fat (r^2^ = −0.78) and β2 receptors (r^2^ = −0.85). Our results showed that: (1) chronic fructose overload induced severe baroreflex impairment, i.e. in a similar magnitude to that observed in SAD rats, which is accompanied by cardiometabolic dysfunctions; (2) the compensatory enhancement in parasympathetic function in SAD rats submitted to fructose intake may point out the possibility of use of approaches that improve vagal function as therapeutic target to attenuate fructose-induced cardiometabolic dysfunctions.

## Introduction

Metabolic syndrome (MetS) is a multi-factorial cluster of metabolic and cardiovascular derangements and impaired molecular mechanisms that plays an important role in cardiovascular disease. MetS is thought to increase overall mortality by 50% and cardiovascular death by 150% compared to subjects without the condition^1,2^. Therefore, a better understanding of not only the altered metabolic and cardiovascular function resulting from MetS but also the mechanisms that mediate these alterations is of importance.

MetS, which may be experimentally induced by chronic and excessive fructose intake^3^, is characterized by several metabolic derangements, such as weight gain, dyslipidemia, increased blood pressure, and insulin resistance^4^, as well as autonomic dysfunction, e.g., reduced heart rate variability and increased blood pressure variability^5^, decreased parasympathetic activity^6^ and baroreflex impairment^7^. It has previously been demonstrated that autonomic dysfunction occurs before metabolic alterations in fructose-fed experimental animals^8^. This suggests that the cardiovascular autonomic nervous system plays a key role as a modulator of fructose-induced changes. Indeed, parasympathetic and baroreflex dysfunction have been associated with fructose-induced metabolic (Brito *et al*.^6^; Moraes-Silva *et al*.^7^), and oxidative stress^9^ alterations.

The baroreflex is a moment-to-moment autonomic modulator of the cardiovascular system, and baroreflex impairment has been considered a major risk marker for cardiovascular disease and mortality^10^. Chronic sinoaortic denervation (SAD), an experimental procedure that abolishes baroreflex control, is used to study baroreflex-induced feedback pathways due to its key role in sympathetic and parasympathetic nervous system modulation^11,12^. Interestingly, twenty days after baroreceptor deafferentation, the parasympathetic efferent pathway undergoes functional and cellular adaptations, leading to increased vagal responsiveness^12^. Reinforcing the role of vagal function in homeostasis, its anti-inflammatory potential has been demonstrated in several studies^13–15^. Further investigation of how the baroreflex modulates autonomic feedback pathways may help understanding the onset and progression of fructose-induced MetS.

Since MetS compromises the baroreceptor reflex^16^, not only is adequate autonomic-controlled buffering of blood pressure oscillations impaired but also the regulation of different metabolic parameters. Therefore, we aimed to demonstrate that baroreflex-mediated autonomic control of the circulation is a pivotal mechanism in cardiovascular, inflammatory and metabolic alterations induced by chronic and excessive fructose intake.

## Results

### Sinoaortic denervation induces baroreflex impairment

SAD efficacy was demonstrated by the significant impairments measured in baroreflex activity and sensitivity in the experimental groups when compared to the Ctrl group (Table 1). Groups which underwent SAD surgery displayed marked decreases in alpha index, in the total number of pressure change events (total ramps), in the total number of spontaneous baroreflex responses (BR ramps), and in bradycardia and tachycardia reflex indexes. Interestingly, the baroreflex deficit observed in the Fruc group was similar to that observed in denervated rats (Table 1).

**Table 1:** Baroreflex measurements.

	Ctrl	Fruc	Den	Den-Fruc	p
Alpha LF (ms/mmHg)	0.96 ± 0.06	0.70 ± 0.05*	0.62 ± 0.09*	0.61 ± 0.05*	0.0009
Total ramps	812.50 ± 122.87	329.60 ± 43.54*	360.50 ± 54.49*	411.50 ± 47.45*	0.0002
BR ramps	263.90 ± 45.03	33.20 ± 6.90*	27.75 ± 5.63*	27.38 ± 4.36*	0.0001
BEI	0.32 ± 0.02	0.10 ± 0.01*	0.08 ± 0.01*	0.07 ± 0.01*	0.0001
Gain-up	3.83 ± 0.29	1.73 ± 0.16*	1.82 ± 0.41*	1.89 ± 0.15*	0.0001
Gain-down	2.74 ± 0.19	1.50 ± 0.11*	1.44 ± 0.17*	1.52 ± 0.20*	0.0001
Gain-total	3.40 ± 0.23	1.68 ± 0.14*	1.60 ± 0.22*	1.74 ± 0.16*	0.0001

Baroreflex activity and sensitivity measured by alpha index and sequence method. Control (Ctrl), Fructose overload (Fruc), sinoaortic denervation (Den), and Den associated with fructose overload (Den-Fruc). *p < 0.05 when compared to Ctrl by Bonferroni post-hot. ANOVA represented by p on graphic.

### Fructose overload induces metabolic syndrome

Measurements of body weight, abdominal adipose tissue and metabolic parameters, such as lipid profile and glycaemia are shown in Table 2. As expected, Fruc rats presented increased body weight and abdominal adipose tissue weight compared to the Ctrl rats. The Den group presented decreased body weight compared to Ctrl and Fruc groups.However, the Den-Fruc group displayed augmented body weight compared to Ctrl and Den groups. While Den rats presented abdominal adipose tissue weight comparable to Ctrl, the Den-Fruc group increased abdominal fat in 30% compared to Ctrl and 54% when compared to Den groups.

**Table 2:** Weight and metabolic profile.

	Ctrl	Fruc	Den	Den-Fruc	p
Body weight (g)	506.32 ± 2.33	569.05 ± 5.25*	459.20 ± 4.40*^#^	550.80 ± 7.80*^$^	0.0001
Abdominal fat (g)	6.28 ± 0.63	12.42 ± 0.81*	5.33 ± 0.52	8.21 ± 0.63^#$^	0.0001
Glycemia (mg/dl)	80.80 ± 4.15	93.20 ± 3.49	86.60 ± 2.66	89.40 ± 2.75	0.0909
Total cholesterol (mg/dl)	131.25 ± 8.31	240.36 ± 10.20*	128.34 ± 7.65	153.36 ± 12.54^#^	0.0001
Triglycerides (mg/dl)	112.36 ± 4.25	168.25 ± 6.63*	123.65 ± 8.32	142.23 ± 7.35*	0.0001
kITT (%/min)	3.48 ± 0.13	2.32 ± 0.11*	3.78 ± 0.17	3.38 ± 0.11^#^	0.0001

Measurements of body weight, abdominal fat, fasting glycaemia, total cholesterol, triglycerides, and constant of insulin tolerance test (kITT) at the end of the 90-day follow up. Control (Ctrl), Fructose overload (Fruc), sinoaortic denervation (Den), and Den associated with fructose overload (Den-Fruc). *p < 0.05 vs. Ctrl; ^#^p < 0.05 vs. Fruc; ^$^p < 0.05 vs. Den by Bonferroni post-hot. ANOVA represented by p on graphic.

In terms of the metabolic profile, the fasting glycaemia were similar among the four groups. Fructose consumption caused hypercholesterolemia and hypertriglyceridemia in Fruc compared to Ctrl group. However, the Den-Fruc group presented just an increase in triglyceride levels compared to Ctrl group. Moreover, total cholesterol was reduced in Den-Fruc group compared to the Fruc group (Table 2). In addition, a reduction in kITT index was observed in Fruc group in comparison with the Ctrl and Den-Fruc groups, indicating insulin resistance.

### Autonomic nervous system adaptations are differently impacted by SAD and fructose overload

**Figure 1:**
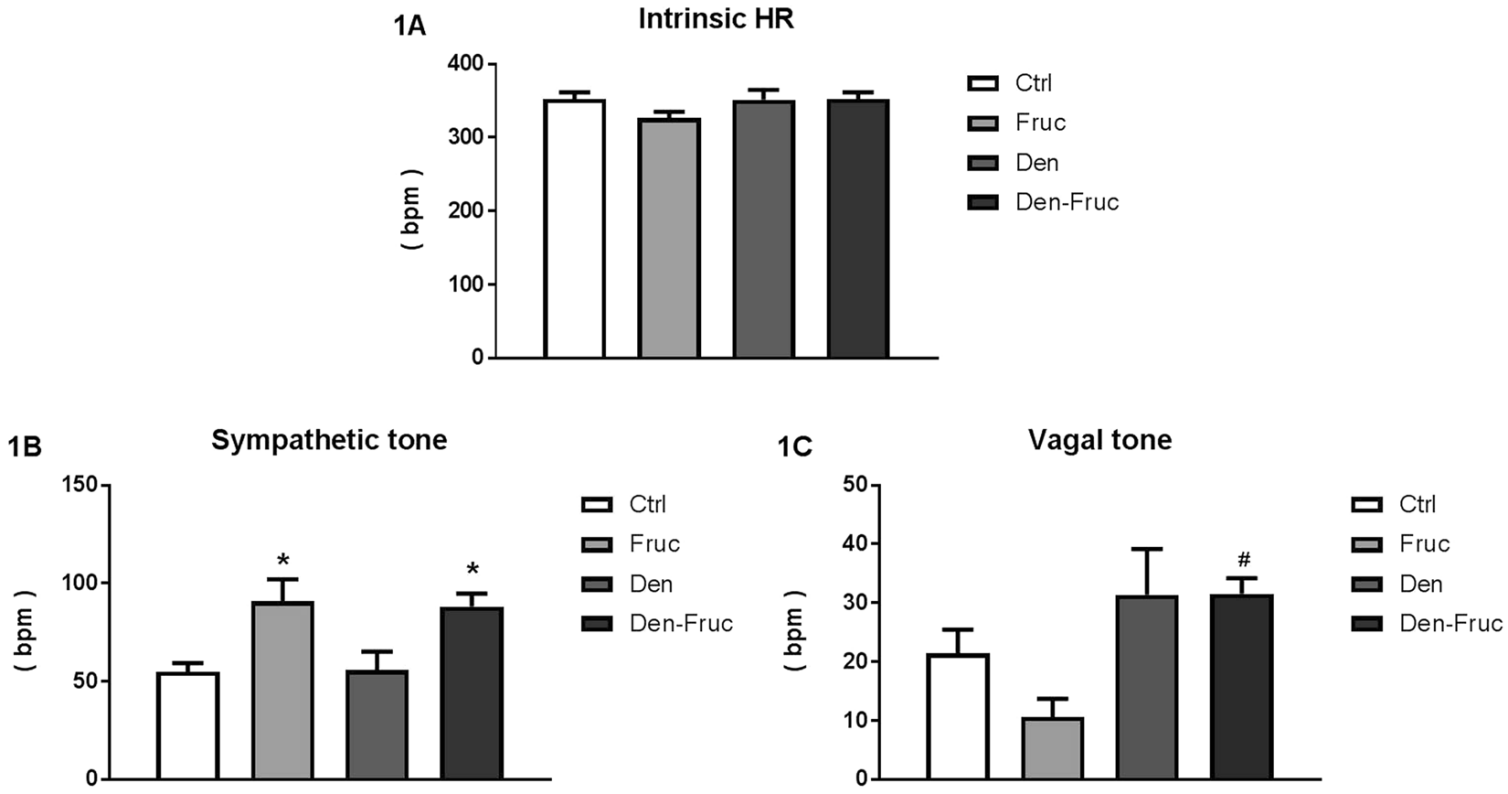
Autonomic blockade. Intrinsic heart rate (**1A**), sympathetic (**1B**) and vagal tone (**1C**) obtained by autonomic blockade with propranolol and methylatropine, respectively. Control (Ctrl), Fructose overload (Fruc), sinoaortic denervation (Den), and Den associated with fructose overload (Den-Fruc). *p < 0.05 vs. Ctrl; ^#^p < 0.05 vs. Fruc by Bonferroni post-hoc, ANOVA (**1A** p = 0.240; **1B** p = 0.006; **1C** p = 0.018).

Selective blockers were used to evaluate autonomic tonus to the heart. Propranolol, a β1 receptor blocker, was used to interrupt sympathetic effector response, while methylatropine, a muscarinic antagonist, was used to inhibit parasympathetic activity. The combination of these two drugs affected intrinsic heart rate, which was not different between studied groups (Fig. 1A). The fructose groups showed increase in sympathetic tone when compared to Ctrl group (Fig. 1B). As for parasympathetic tone, we found that SAD was able to increase it; however, this increase was only statiscally significant when comparing Den-Fruc to Fruc group (Fig. 1C).

Regarding hemodynamics, the Den-Fruc group displayed a higher heart rate compared to all other groups. Systolic blood pressure increased in both groups treated with fructose compared to Ctrl rats, and was higher in Den-Fruc than in Ctrl and Den animals (Table 3).

**Table 3:** Hemodynamics, heart rate variability and systolic blood pressure variability.

	Ctrl	Fruc	Den	Den-Fruc	p
HR (bpm)	353.84 ± 4.69	351.53 ± 4.08	367.40 ± 7.94	399.07 ± 9.78*^#$^	0.0001
VAR PI (ms^2^)	135.13 ± 16.39	100.04 ± 10.13	45.65 ± 8.92*	73.25 ± 17.20*	0.0005
LF PI (ms^2^)	6.25 ± 0.98	14.99 ± 0.93*	2.03 ± 0.46*	2.85 ± 0.52*^#^	0.0001
HF PI (ms^2^)	13.83 ± 0.95	12.57 ± 1.80	7.14 ± 0.64*	8.33 ± 0.95*	0.0013
LF PI (%)	11.40 ± 0.82	27.63 ± 0.83*	9.63 ± 1.19	10.75 ± 0.67^#^	0.0001
HF PI (%)	28.80 ± 2.18	22.04 ± 1.74	43.13 ± 5.03*	39.13 ± 4.39^#^	0.0003
LF PI (nu)	29.60 ± 2.46	56.15 ± 2.07*	21.00 ± 3.69*	23.63 ± 2.69^#^	0.0001
HF PI (nu)	70.40 ± 2.46	43.85 ± 2.07*	79.00 ± 3.69*	76.38 ± 2.69^#^	0.0001
LF/HF	0.48 ± 0.07	1.33 ± 0.11*	0.31 ± 0.07*	0.34 ± 0.05^#^	0.0001
SBP (mmHg)	120.80 ± 1.07	143.05 ± 3.21*	122.11 ± 5.09	136.68 ± 3.66*^$^	0.0001
VAR SBP (mmHg^2^)	42.10 ± 3.16	40.30 ± 2.43	106.22 ± 24.71*	180.75 ± 40.34*^#^	0.0001
LF SBP (mmHg^2^)	6.54 ± 0.57	9.37 ± 1.06*	5.23 ± 0.75	7.77 ± 1.30	0.0260

Time and frequency domain of heart rate and blood pressure variability. Heart rate (HR), variance of pulse interval (VAR PI), square root of differences between consecutives PI intervals (RMSSD), low frequency band of pulse interval (LF PI), including absolute, percent and normalized units, high frequency of pulse interval (HF PI) including absolute, percent and normalized units, sympathovagal balance (LF/HF), systolic blood pressure (SBP), variance of SBP (VAR SBP) and low frequency band of SBP (LF SBP). Control (Ctrl), Fructose overload (Fruc), sinoaortic denervation (Den), and Den associated with fructose overload (Den-Fruc). *p < 0.05 vs. Ctrl; ^#^p < 0.05 vs. Fruc; ^$^p < 0.05 vs. Den by Bonferroni post-hot. ANOVA represented by p on graphic.

Blood pressure variability was increased in Den when compared to Ctrl group, and was even greater in the Den-Fruc group. However, only the Fruc group demonstrated increased vascular sympathetic modulation (LF SBP) (Table 3).

Considering heart rate variability, SAD (Den and Den-Fruc vs. Ctrl group) promoted a reduction in total variability, represented by VAR PI; and consequently a reduction in the specific interest spectrum band, absolute LF and HF PI (Table 3). However, when we analyzed the representative percentage of total modulation, it became clear that fructose ingestion (Fruc group) led to increased sympathetic modulation to the heart (LF PI) compared to the Ctrl group. This alteration was not observed in Den-Fruc group. It should be emphasized that Fruc group presented a decrease in parasympathetic modulation (HF PI, nu) compared to Ctrl group. Moreover, SAD resulted in an elevation in parasympathetic modulation to the heart (Den and Den-Fruc vs. Ctrl group). Taken together, these findings indicate an increased sympathovagal balance in the Fruc group and decreased sympathovagal balance in both Den and Den-Fruc when compared to Ctrl group (Table 3).

**Figure 2:**
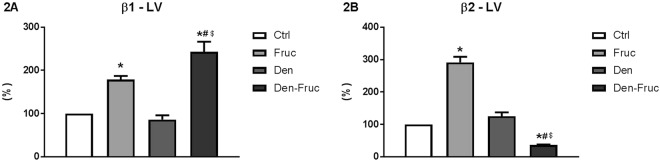
β Receptors expression. Quantification of β1 (**2A**) and β2 (**2B**) receptors in the left ventricle (LV). Percentage of change compared to control group. Control (Ctrl), Fructose overload (Fruc), sinoaortic denervation (Den), and Den associated with fructose overload (Den-Fruc). *p < 0.05 vs. Ctrl; ^#^p < 0.05 vs. Fruc; $p < 0.05 vs. Den by Bonferroni post-hoc, ANOVA (**2A** p < 0.001; **2B** p < 0.001).

Comparing β adrenergic receptor expression in the left ventricle between groups, fructose overload (Fruc group) promoted an increase in both β1 and β2 receptors compared to Ctrl group, while the association between SAD and fructose ingestion (Den-Fruc group) resulted in a significant increase in β1, along with a decrease in β2 receptors in the left ventricle (Fig. 2).

### Fructose overload and/or SAD induced inflammation in cardiac tissue

**Figure 3:**
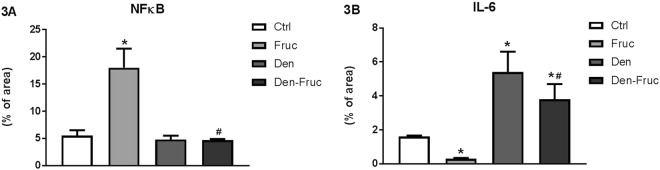
Cardiac immunohistochemistry. Nuclear factor kappa B (NFκB) (**3A**) and interleukin-6 (IL-6) (**3B**) by immunohistochemistry in cardiac tissue. Control (Ctrl), Fructose overload (Fruc), sinoaortic denervation (Den), and Den associated with fructose overload (Den-Fruc). *p < 0.05 vs. Ctrl; ^#^p < 0.05 vs. Fruc by Bonferroni post-hoc, ANOVA (**3A** p = 0.005; **3B** p < 0.001).

**Figure 4:**
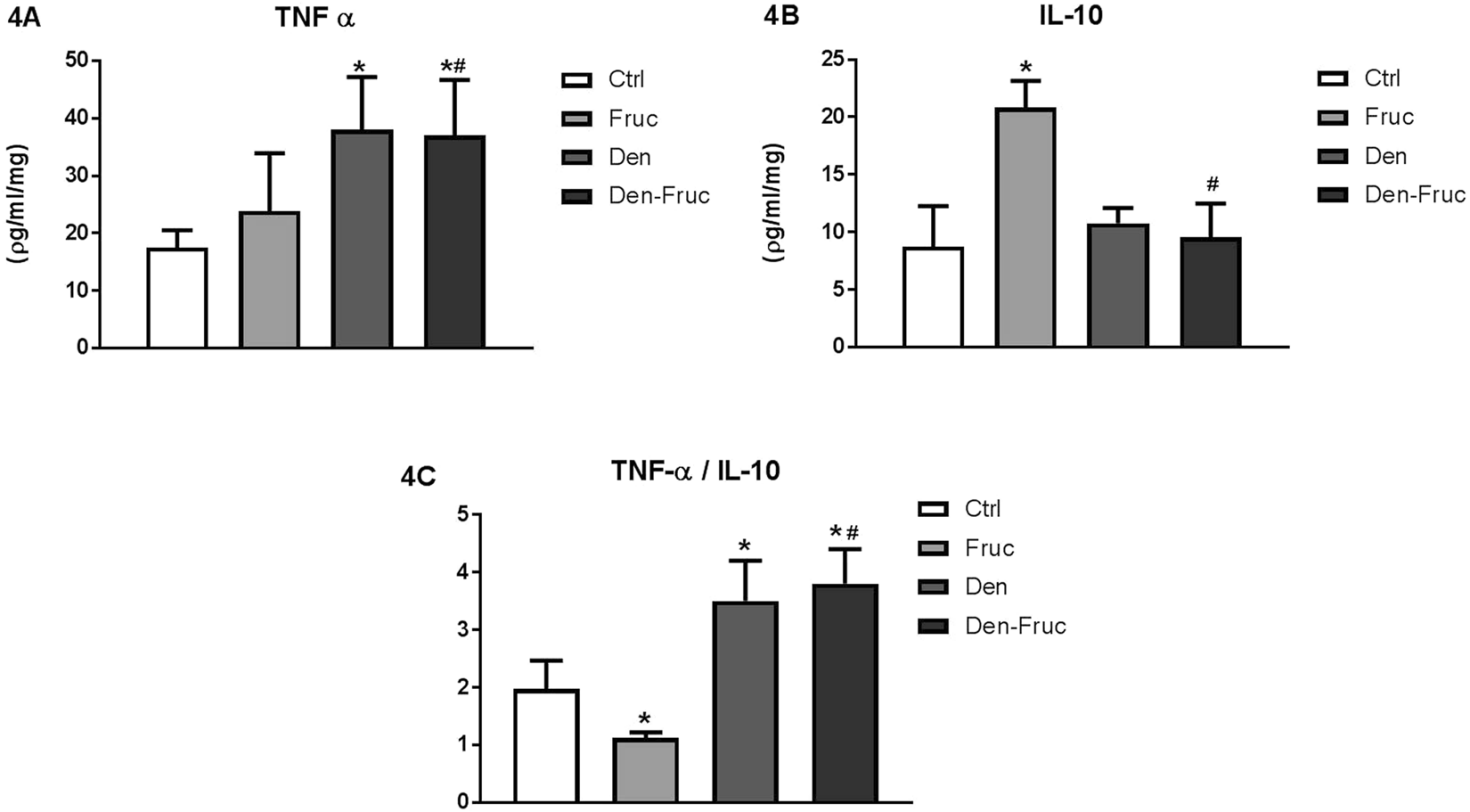
Cardiac inflammation. Tumor necrosis factor alpha (TNFα) (**4A**) interleukin-10 (IL-10) (**4B**) and the TNFα/IL-10 ratio (**4C**) by ELISA in cardiac tissue. Control (Ctrl), Fructose overload (Fruc), sinoaortic denervation (Den), and Den associated with fructose overload (Den-Fruc). *p < 0.05 vs. Ctrl; ^#^p < 0.05 vs. Fruc by Bonferroni post-hoc, ANOVA (**4A** p = 0.018; **4B** p = 0.039; **4C** p = 0.023).

Examining the cardiac inflammatory profile, Fruc rats showed an increase in NFkB and IL-10, with a reduced IL-6 and TNFα/IL-10 ratio compared to Ctrl rats. Both denervated groups, however, demonstrated increased TNFα and IL-6, without any changes in NFkB and IL-10 values, which in turn increased the TNFα/IL-10 ratio compared to Ctrl group (Figs 3, 4).

### Association analyses

To determine to what extent studied parameters were co-associated, correlation analyses were performed (Table 4). Increased sympathetic tone was positively correlated with increases in the cardiac β2 receptor population. Interestingly, this event was more evident in the Den group (r^2^ = 0.93), while in Den-Fruc group the correlation was negative. On the other hand, vagal tone was negatively correlated with cardiac β2 receptor density. Once more, this correlation was stronger in Den and Den-Fruc groups. Moreover, higherincreased insulin sensitivity was correlated with an increase in cardiac parasympathetic modulation, especially in Den rats. This can be seen in Table 4, which shows that the higher the insulin sensitivity, the lower the sympathovagal balance.

**Table 4:** Association studies.

	All	Ctrl	Fruc	Den	Den-Fruc
Sympathetic Tone and β2	0.3375	0.6574	0.6871	0.9246	−0.9650
Vagal Tone and β2	−0.6325	−0.6190	−0.4857	−0.9434	−0.8569
kITT and HF PI (%)	0.7572	0.8128	0.1158	0.9454	0.2780
KiTT and LF/HF	−0.7829	−0.1536	−0.4928	−0.1257	0.1356
HF PI % and Abdominal Fat	−0.7766	0.2897	−0.0193	0.1361	−0.2943

Correlation analysis (r^2^) between the following parameters: cardiac sympathetic tone and cardiac β2 receptors expression, cardiac vagal tone and cardiac β2 receptors expression, insulin sensitivity (kITT) and percentage of cardiac vagal modulation (HF), insulin sensitivity (kITT) and cardiac sympathovagal balance (LF/HF), and percentage of cardiac vagal modulation (HF) and abdominal fat. All groups together (All), Control (Ctrl), Fructose overload (Fruc), sinoaortic denervation (Den), and Den associated with fructose overload (Den-Fruc).

## Discussion

The central aim of this study was to investigate the role of the baroreflex as a modulating mechanism of fructose-induced adaptations (and MetS). SAD surgery was effective in this regard: both Den and Den-Fruc groups presented significantly diminished heart rate responses to blood pressure changes, as evidenced by the sequence method and the spontaneous baroreflex evaluated by alpha index^17,18^.

Fructose overload was also effective in inducing a metabolic syndrome phenotype. A range of effects of fructose intake in animal models have been described according to concentrations, exposure time, administration, gender, age, species and others^19^. In our study, 90 days of 10% fructose ingestion in drinking water increased body weight, abdominal fat, total cholesterol, triglycerides (Table 2) and systolic blood pressure (Table 3), while decreasing kITT, thus characterizing MetS.

Interestingly, fructose consumption reduced baroreflex sensitivity and activity similarly to (Table 1) SAD. Our group has already demonstrated that fructose overload is capable of promoting baroreflex impairment^7^. In the present study, we found that this deficiency in Fruc rats is similar to that caused by SAD surgery, suggesting that long-term excessive fructose intake can be as harmful as SAD to baroreflex function.

Altered autonomic responses to fructose and SAD were the main observations of the present study. Based on the results of autonomic blockade with propranolol and methylatropine, sympathetic tone was increased in both groups treated with fructose (Fig. 1B). This was expected, since sympathetic hyperactivity is correlated with increased adipose tissue, dyslipidemia and hypertension^20,21^. In addition, fructose may act directly on the central nervous system, thus increasing sympathetic activity^22^. Additionally, the Fruc group demonstrated increased sympathetic modulation in both absolute and percent values compared to Ctrl (Table 3). This resulted in increased sympathovagal balance, increased peripheral sympathetic modulation and blood pressure. This elevation in blood pressure may be linked not only to sympathetic hyperactivity but also to baroreflex dysfunction^23^.

Isolated SAD produced a reduction in the total modulation (VAR IP) and a consequent decrease in the absolute band of sympathetic and parasympathetic modulation. Additionally, we found an increased dominance of parasympathetic modulation in the Den group, thus reducing the sympathovagal balance. Although there was no change in SBP, VAR SBP was severely augmented, as is characteristic of this model^24,25^.

Surprisingly, when Fruc and SAD were experimentally combined, alterations caused by fructose consumption, such as increased abdominal fat, cholesterol, and insulin resistance were attenuated, together with smaller increases in body weight and SBP compared to the Fruc only group. Therefore, we may ask, how can SAD attenuate fructose-induced metabolic derangements?

Despite the fact that there is a parasympathetic hyperresponsiveness after chronic SAD in control rats^12^, we firstly believed that, after 90 days, SAD-induced impairments would already be established in Fruc-Den rats, as observed in other SAD-associated conditions^26–28^. Conversely, we observed that parasympathetic compensation was still present, together with attenuation in some fructose-induced alterations. This finding suggests that severe baroreflex impairment in fructose-fed rats may induce a vagal compensatory mechanism to modulate cardiometabolic impairments caused by chronic fructose overload.

Another relevant detail is that vagal function is associated with kITT, as shown by Kreier *et al*.^29^ and reinforced by the correlation presented in this study (r^2^ = 0.76). Sinaiko *et al*.^30^ have demonstrated in humans that insulin sensitivity at 13 years of age was predictive of metabolic disorders found 6 years later. Higher rates of obesity, increased blood pressure, cholesterol and triglycerides were found in this population. The same effect may be observed in our animals, as both denervated groups presented insulin sensitivity values similar to the control group. This may have contributed to the attenuation of the metabolic effects promoted by fructose in the Den-Fruc group, which displayed a reduction of abdominal fat, cholesterol and blood pressure.

While the relationship between insulin resistance and vagal activity may justify the attenuation of metabolic changes, the interaction between cardiac inflammation and beta adrenoceptors with vagal activity may account for the other observed results. Isolated denervation did not alter gene expression of β1 and β2 receptors, in accordance with the desensitization of β adrenoceptors after long exposures to enhanced sympathetic activity^31^. Conversely, in the Fruc group, secondary to increased sympathetic modulation and tonic effect to the heart, an increase in β1 receptor expression is needed to ensure autonomic responsiveness^32^. As a compensatory response, there was also an increase in β2 receptor gene expression, and this may be related to the prevention of the increase in heart rate in these animals and to the reduction, although not significant, of intrinsic heart rate, thus preventing changes in heart rate variability. Reinforcing the role of sympathetic tonus in this response, a positive correlation between this parameter and β2 receptors was found for Fruc rats (r^2^ = 0.68).

In the combined SAD and fructose group (Den-Fruc), increased heart rate was observed compared to all other groups (Table 3). Since this group demonstrated a greater sympathetic tone to the heart compared to other groups without reducing intrinsic heart rate, as did the Fruc group, this change was expected. The Den-Fruc group also showed a significant increase in the expression of β1; however, as the vagal tone and modulation were already elevated, there was no compensatory response mediated by β2 receptors. The increase in cardiac β1 receptor expression may also explain this heart rate increment in Den-Fruc, since it is well-known that β1 receptors are more important for positive chronotropism than β2 receptors^33^. We should note that both sympathetic and vagal tonus demonstrated strong negative correlations (r^2^ < −0.85) with β2 receptor concentration for Den-Fruc rats, showing that when both sympathetic and vagal tonus are increased, the gene expression of β2 receptors is decreased.

In this regard, the gene expression of cardiac β2 receptors may play a role in the cardiac inflammatory profile. Distinct inflammatory responses among the groups were found in the cardiac muscle. Fructose consumption seems to lead to a profile of inflammation related to metabolic residues. The increase in NFkB indicates tissue injury, which may be associated with the oxidative stress caused by residues in fructose metabolism^34^. This may be due to the preferential use of fructose as the main energy source, rather than glucose^35^. There was also an increase in the anti-inflammatory cytokine IL-10, which has been found to have a direct association with increased NFkB^36^, thus indicating an attempt to maintain the balance between pro and anti-inflammatory cytokines. This condition was observed when the TNFα/IL-10 ratio was analyzed, and a very significant reduction of this inflammatory balance was found. Another contributing factor may be the increase in cardiac β2 receptors in Fruc animals, since its activation through the parasympathetic nervous system improved the anti-inflammatory profile^15^.

Conversely, denervated groups had a different inflammatory pattern. We observed an impressive increase in IL-6, indicating the activation of immune system cells. This probably occurs due to a greater infiltration of these cells caused by increased blood pressure variability^37^. It is known that the higher the blood pressure variability, the higher the risk of target organ damage, regardless of the blood pressure values^38^, thus generating a greater inflammatory response. In fact, both SAD groups demonstrated an increase in the inflammatory profile, as demonstrated by the enhanced TNFα/IL-10 ratio. It should also be stressed that this inability to increase the anti-inflammatory response may be associated with the fact that the NFkB values remained unchanged. In addition, there is evidence of a weak relationship between parasympathetic and the inflammatory profile in these groups. Despite the increased vagal tone and vagal modulation values, there was no improvement in the anti-inflammatory profile in our hands. This may be due to the reduction in β2 receptors, as demonstrated in the Den-Fruc group.

In summary, the main results from our experiments are the following: (1) chronic fructose overload induced changes in cardiometabolic parameters accompanied by severe baroreflex impairment, i.e. in a similar magnitude to that observed in chronic denervated rats; (2) chronic SAD enhances parasympathetic activity, corroborating previous studies in control rats^12^; (3) this vagal enhancement is also present when SAD is concomitantly associated with chronic fructose intake, and is strongly associated with favorable cardiac and metabolic profile changes. Nonetheless, it does not mean that SAD itself is beneficial in metabolic syndrome; actually, it shows that, in the presence of a metabolic pathological stimulus, such as fructose overload, the parasympathetic compensation induced by baroreflex deficiency may be longer and to a greater magnitude than what was observed in the study of Soares *et al*.^12^, in which the increased vagal responsiveness was present 20 days after SAD. The vagal attempt to counterbalance the systemic metabolic alterations induced by fructose in the presence of complete baroreflex dysfunction may point out the possibility of use of approaches that improve vagal function as therapeutic target to attenuate fructose-induced cardiometabolic dysfunctions. Moreover, our findings reinforce the role of the baroreflex in physiological and pathological adaptations, and this may be a target mechanism in the onset and development of MetS.

## Methods

### Bioethical Statement

We declare that all methods used in this protocol were carried out in accordance with relevant guidelines and regulations. All experiments were carried out after approval by Ethical Committee for Animal Use from the University of Sao Paulo Medical School under protocol number 274/11 of 07/13/2011.

### Animal model and groups

We used male Wistar rats (*Rattus norvegicus*) weighing around 150 g at the beginning of the protocol. Animals were kept in the Heart Institute (InCor) Animal Shelter, four specimens per cage with free movement, and access to food and water *ad libitum*. The temperature was kept at 20–22 °C and the light-dark cycle was 12–12 h.

MetS was induced by fructose overload (100 g/l in drinking water)^39^. Our four experimental groups were assigned as follows: the control group (Ctrl), monitored for 90 days (n = 10); the fructose group (Fruc), receiving 10% solution of fructose from the beginning to the end of the 90-day follow-up period (n = 10); the sinoaortic denervation group (Den), which underwent a surgical removal of the baroreceptor afferent fibers at day 1 and was monitored for 90 days (n = 8); and the sinoaortic denervated fructose group (Den-Fruc), in which rats were denervated at day 1 and received the 10% fructose solution for 90 days.

### Surgical procedures

For sinoaortic denervation surgery, animals were anesthetized with isoflurane (1.5% on O_2_ flow). They were placed in supine position and an incision was made in the neck, exposing the carotid artery. The baroreceptor fibers were identified and cut at sympathetic nerve, while the Hering nerve and laryngeal nerve were cut at carotid bifurcation. This procedure was performed on both sides, as described by Krieger *et al*.^17^ Krieger^17^.

At the end of the 90-day protocol, animals underwent catheterization of the femoral artery and vein. Once more, they were anesthetized with isoflurane (2.5% on O_2_ flow) due to their weight gain. Polyethylene cannulas were inserted into the femoral artery (PE-10, with internal diameter of 0.1 mm) and into the femoral vein (PE-50, with internal diameter of 0.5 mm). Both cannulas were connected to a larger tube (Tygon, with an internal diameter of 0.8 mm). The cannulas were filled with heparinized saline and fixed in the femoral vessels using cotton suture wire, and the extremity with the larger diameter was externalized in the dorsal cervical area and fixed to the skin using cotton suture wire. The externalized portion of the cannula was kept closed by using stainless steel pins. After surgery, animals were treated with a single injection of penicillin (Benzetacil, Fontoura- Wyeth, 30.000 U/kg) and analgesic (Dipyrone, 0.35 g/kg).

### Hemodynamics

After surgical preparation, the animals were placed in individual cages with free access to food and water (or water containing 10% fructose) for 48 hours for recovery. On the first day of the experiment, the rats were connected to the computerized acquisition systems by connecting the arterial cannula to a polyvinyl tube (Tygon), which was attached to an electromagnetic transducer (Kent Instruments). This transducer was linked to an amplifier (General Purpose Amplifier-Stemtech, Inc.) and connected to a computer with an analog to digital converter board (DataQ/Windaq, sampling rate of 2000 Hz per channel), which allows real-time data acquisition of pulse waves and heart rate and posterior software analysis^11^.

The animals remained connected for half an hour to the system for adaptation, and signal acquisition was then started. A basal period was recorded for 30 minutes. Pharmacological autonomic blockade was performed to evaluate sympathetic and parasympathetic tonus. For this purpose, one injection of propranolol (8 mg/kg) and/or methylatropine (4 mg/Kg) was randomly administrated in the cannula attached to the femoral vein. After a short period wait for drug action, a new recording was performed for 10 minutes. Twenty-four hours after the first blockade, the procedure was repeated in reverse order of the drug-delivery. Therefore, we managed to isolate the propranolol and methylatropine effects on intrinsic heart rate, enabling us to measure the parasympathetic and sympathetic tonus to the heart^40^.

Analysis of blood pressure signals was performed using an algorithm implemented in Windaq/DataQ, which was associated with an acquisition system. This software allowed the detection of the maximal value in the blood pressure curve, beat to beat, providing the values of systolic blood pressure (SBP). Heart rate was determined from the pulse interval (PI) between two systolic peaks.

### Heart rate and blood pressure variability

Heart rate variability was measured by linear methods in time and frequency domains by using the Cardioseries® v2.4 software. Temporal series of PI and SBP (30 minutes of baseline recording) were analyzed in time domain, obtaining the total variance of PI (VAR PI) and the variance of SBP (VAR SBP). Mean square root of differences between consecutives PI (RMSSD) was also evaluated.

For the frequency domain, the interpolated waves of these same baseline periods were divided into segments of 512 beats, with an overlap of 50%, and were processed by Fast Fourier Transform. One spectrum was obtained for each segment and the oscillatory components were quantified in two different frequencies: low frequency (LF) from 0.20 to 0.75 Hz and high frequency (HF) from 0.75 to 3.00 Hz. The results are represented by absolute values (ms^2^ and mmHg^2^), the percentage of total spectrum (%) and normalized units (nu) (percentage of LF and HF bands only). The very low oscillations (<0.20 Hz) were considered non-stationary^7^.

### Baroreflex

Baroreflex activity and sensitivity were measured in two different ways using the Cardioseries® v2.4 software: the alpha index of LF band, and the spontaneous baroreflex by the sequence method. The first one was calculated by the square root of the ratio between the absolute value of LF PI by the absolute value of LF SBP, correlating the absolute values of LF for the heart with absolute LF for the vessels, when they were consistent with each other. The second one required finding 4 points with consecutive increases or decreases in blood pressure associated with a consecutive 4 points of decreasing or increasing heart rate, respectively. We evaluated the total number of events (total ramps), the number of events presenting a heart rate response (BR ramps), the ratio between BR ramps and total ramps (BEI) and the gain, which represents the baroreflex sensitivity for increasing or decreasing blood pressure^41^.

### Metabolic profile

After 90 days and before the cannulation procedure, the animals were weighed and underwent 4 hours of fasting. Then, a small cut on the tail tip was performed to collect a few drops of blood. Glycaemia (Accu-check, Roche®), total cholesterol and triglycerides (Accutrend, Roche®) were measured using reactive tapes. The insulin tolerance test (ITT) was performed by injecting 0.75 U/kg of insulin on the tail vein. Glycaemia was measured at 0, 4, 8, 12, and 16 minutes after insulin administration. These values were used to calculate the glycaemia decay by constant of insulin tolerance test (kITT)^42^. After all procedures, the animals were euthanized by decapitation. The abdominal adipose tissue and hearts were collected and weighed.

### PCR analysis of β1 and β2 receptors gene

Cardiac tissue was separated between atrium and ventricles and 100 mg of the left ventricle was treated with 1.0 ml of Trizol® reagent (InvitrogenTM). The samples were homogenized and total RNA was extracted. Concentrations were measured (Thermo Scientific) at a wavelength of 260–280 nm. We obtained an aliquot of 3 µg of each RNA sample and treated it with Turbo DNA-Free (Ambion) to exclude possible contamination by DNA. Reverse transcription was carried out using SuperScriptTM II Reverse Transcriptase (InvitrogenTM) for proteins of interest. Finally, we ran a real time PCR using β-actin and GAPDH as housekeeping proteins. Quantification was undertaken using the difference between target proteins minus the average of two housekeeping proteins. To compare the results, we normalized the Ctrl group at 100% and created a relation for the other groups.

### Cardiac inflammatory mediators

Cardiac cytokines were quantified in the left ventricle using a commercial ELISA kits from R&D Systems (Minneapolis, MN, USA). We used markers for interleukin 10 (IL-10) and tumor necrosis factor alpha (TNFα). The results are presented as the ratio of marked proteins to the total amount of protein^43^. For interleukin 6 (IL-6) and nuclear factor kappa B (NFκB) measurements, we used histological sections of the left ventricles. We performed a circulatory perfusion of potassium chloride (14 mmol/l), followed by buffered formaldehyde (3.7%). After histological preparation, the slides were incubated with a complex containing primary and secondary antibodies to the proteins of interest^43^. The measurements were performed by colorimetric quantification (Image J®) obtained by 20 photos per slide in a 400× increase.

### Statistics

All data were submitted to D’Agostino and Pearson omnibus normality test. They are expressed as mean and standard error of mean. To determine significance level, we used ANOVA with Bonferroni post-hoc test. Association between variables was evaluated using Pearson’s correlation analysis. P value < 0.05 was considered statistically significant.
